# Comments about the appraisal of systematic reviews in restorative dentistry

**DOI:** 10.12688/f1000research.53117.3

**Published:** 2022-02-23

**Authors:** Kelvin I. Afrashtehfar, Musab H. Saeed

**Affiliations:** 1 Division of Restorative Dental Sciences, Clinical Sciences Department, Ajman University, Ajman, P.O. Box 346, United Arab Emirates; 2 Department of Reconstructive Dentistry and Gerodontology, School of Dental Medicine, University of Bern, Berne, BE, 3010, Switzerland; 3 Centre of Medical and Bio-allied Health Sciences Research, Ajman University, Dubai, City of Gold, United Arab Emirates

**Keywords:** data management, dentistry, evidence-based medicine, evidence-based practice

## Abstract

Adequate adoption of evidence-based practice is deeply rooted in accessing methodological quality and completeness of systematic reviews and meta-analyses reporting. Nonetheless, this assumption might be flawed if the methodological quality assessment has not been properly conducted. Taking the former statement into consideration, this correspondence article encourages the improvement of future tertiary manuscripts, especially in the field of restorative dentistry. Thus, this article addresses an overview of reviews in restorative dentistry as an example of evaluating tertiary evidence for increasing the awareness of reviewers, authors, and readers.

## Comments about the appraisal of systematic reviews in restorative dentistry

Dear respectable Advisory Editors and readers:

The publication by Sarkis-Onofre *et al.* ^1^ “Systematic reviews in restorative dentistry: discussing relevant aspects,” in the *Journal of Esthetic and Restorative Dentistry* in the May 2019 issue was read with great and particular interest. This well-written overview of reviews or systematic review of systematic reviews stated that “This study was not registered in PROSPERO” since PROSPERO indicates that “Reviews of methodological issues need to contain at least one outcome of direct patient or clinical relevance in order to be included in PROSPERO.” Interestingly, despite the fact that the above referenced tertiary study falls in the PROSPERO's review of reviews category, it was neglected to being classified as such. Additionally, if PROSPERO was not an option, the a priori protocol could have been easily registered in another repository. Therefore, the authors' arguments not to register their protocol in PROSPERO are not valid.

Moreover, the authors ^1^ mentioned that the previous version of their review ^2^ has a protocol available upon request. However, their first paper ^2^, which is in a Brazilian University Magazine printed in Portuguese language, does not support the updated version of the review properly since their first version does not consider any protocol in the text.

Their critical appraisal using AMSTAR-2 appears in Table 2. Five out of the 16 included review studies in their review of reviews, had between one to four AMSTAR-2 items referred as “Authors reported different information by e-mail however, it was not found in the article.” This reporting method may not be the most scholarly or safest to present their findings, especially when the authors of their included review studies kindly accepted to provide further clarification about their methodology.

Particularly, the first author of Afrashtehfar *et al.* ^3^ “Failure rate of single-unit restorations on posterior vital teeth: a systematic review” regretted the online communication with their corresponding author when he was requested to provide further information. Perhaps Sarkis-Onofre *et al.* should have dedicated more time to conduct an adequate assessment ^4^. For example, their unfavorable categorization of the AMSTAR-2 items 4, 7, and 16 for Afrashtehfar *et al.* ^3^ may be mistaken. A comprehensive literature search (item 4) can be considered in the former paper ^3^ since it searched for published papers for over 20 years with no language restriction, using four electronic databases (including Embase and Medline via Ovid) and displaying each search strategy in the Appendix section. Additionally, the review hand searched eight journals and also screened manually in the reference list of all identified relevant primary studies and related secondary studies ^3^. Next, the list of excluded primary studies and justifications (item 7) were provided in Supplemental Table 6 (i.e., full-text excluded articles and reasons for exclusion). Regarding any potential sources of conflict (item 16), it is well-stated on the first page of the review that this study was “Supported in part by a Knowledge Transfer Grant from the Network for Oral and Bone Health Research.” Additionally, there is a section at the end of the paper for Acknowledgements where librarians and statisticians were thanked for their consultancy ^3^.

Moreover, the search and eligibility criteria for Sarkis-Onofre *et al.* ^1^ were systematic reviews that met PRISMA-P, including adults over 18 years of age with direct composite resin restoration in posterior teeth compared with other materials or techniques used in posterior teeth regardless of the outcome up to October 15 ^th^, 2017. However, some articles that fully suited their inclusion criteria were not included. For example, Afrashtehfar *et al.* ^5^ “Failure of single-unit restorations on root filled posterior teeth: a systematic review” was not included despite being available from November 21 ^st^, 2016. Therefore, their search strategy and their search conduct (including the elimination of duplicates) ^6^, as well as screening ^7^, raise some serious methodological concerns ^8^.

Their overview of reviews has a collaboration with well-known evidence-based medicine experts from Canada, Tricco and Moher ^9^, which usually rely on the talent from their research team for screening and assessing the literature.

After a brief analysis, this letter encourages the improvement of future synthesis of studies including their quality assessment to:

▪ Address clarification with authors of potentially included studies safely and respectfully to avoid accusation, especially if there is no consensus on the matter from different experts ( *i.e.*, two experts as a minimum) ^10– 15^.
▪ Take the time and effort necessary to assess the review paper of interventions according to AMSTAR-2 ^4^. At least two experts in the field should also determine this instead of two research trainees.
▪ Spend sufficient time with expert librarians to develop an adequate search strategy in multiple databases ^3, 5^.
▪ Use a reference manager and do not rely on removing the duplicates by selecting only one category ( *i.e.*, authors' names). Thus, the available categories should be combined to avoid removing records that may share the same publication journal, year, or authors.
▪ A PRISMA checklist ^16, 17^ should be submitted, reporting compliance with each item by indicating the paragraph and page where they can be identified in the review/appraisal. All the required reporting should be included in the quality assessment to ensure transparency and validity.

## Data availability

### Underlying data

No data are associated with this article.
