# Supplementary material for: Comments about the appraisal of systematic reviews in restorative dentistry
Source: F1000Res. 2022 Feb 23;10:442. Originally published 2021 Jun 3. [Version 3] doi: 10.12688/f1000research.53117.3 (PMC8792871; doi:10.12688/f1000research.53117.3)
Supplement: Peer review history

# Peer review: Reviewer response for version 3

**DOI:** 10.5256/f1000research.121499.r124764

**Published:** 2022-02-28

**Authors:** Luca Testarelli, Rodolfo Reda

**Affiliations:** 1 Department of Oral and Maxillofacial Sciences, School of Dentistry, Sapienza University of Rome, Rome, Italy

We believe that the article no longer presents any criticism, it is well written and complete.

Are arguments sufficiently supported by evidence from the published literature or by new data and results?

Yes

Is the conclusion balanced and justified on the basis of the presented arguments?

Yes

Is the rationale for commenting on the previous publication clearly described?

Yes

Are any opinions stated well-argued, clear and cogent?

Yes

Reviewer Expertise:

CBCT, Dental Imaging, Endodontics, Oral Microbiology, Oral Surgery.

We confirm that we have read this submission and believe that we have an appropriate level of expertise to confirm that it is of an acceptable scientific standard.

# Peer review: Reviewer response for version 2

**DOI:** 10.5256/f1000research.79657.r101051

**Published:** 2022-01-26

**Authors:** Spyridon N. Papageorgiou

**Affiliations:** 1 Clinic of Orthodontics and Pediatric Dentistry, Center of Dental Medicine, University of Zurich, Zurich, Switzerland

My comments to the content of the piece have been covered.

Are arguments sufficiently supported by evidence from the published literature or by new data and results?

No

Is the conclusion balanced and justified on the basis of the presented arguments?

No

Is the rationale for commenting on the previous publication clearly described?

Yes

Are any opinions stated well-argued, clear and cogent?

Partly

Reviewer Expertise:

Orthodontics; evidence-based dentistry; systematic reviews

I confirm that I have read this submission and believe that I have an appropriate level of expertise to confirm that it is of an acceptable scientific standard.

# Peer review: Reviewer response for version 1

**DOI:** 10.5256/f1000research.56469.r93586

**Published:** 2021-11-16

**Authors:** Spyridon N. Papageorgiou

**Affiliations:** 1 Clinic of Orthodontics and Pediatric Dentistry, Center of Dental Medicine, University of Zurich, Zurich, Switzerland

I read with interest the abovementioned submission. However, I must say that I am somewhat confused since the author used this piece to comment on inadequacies, as deemed by the author, of a JERD-published overview of reviews that also included one of his SRs. In essence, this piece is not thought of as a standalone paper but is rather aimed at the already published overview like a correspondence with points to be addressed by the authors of the original overview in JERD. Therefore, I believe this would be more appropriate to be submitted as such in JERD and not here (not to mention that it is significantly more probable that it will be read and will be replied upon by the overview authors). Thanks for letting me see this.

 Specific comments

1. Abstract. “accessing” is probably “assessing”.
2. Bear in mind that methodological quality is different from the risk of bias. I’m not sure which of the two you are implying here, but it makes sense that it is the latter.
3. “The improvement of future methodological quality assessment manuscripts”: this needs to be reformulated more succinctly.
4. “Thus, this article addresses a methodological quality assessment about systematic reviews in restorative dentistry by Sarkis-Onofre *et al.* in the May 2019 issue of the Journal of Esthetic and Restorative Dentistry as an example of evaluating appraisals of reviews for increasing the awareness of reviewers, authors, and readers.” Instead of relaying what this piece does, better relay its’ scope/aim and its final recommendations.
5. Text. The cited paper is neither “methodological quality assessment of systematic reviews” (methodological quality assessment is a procedure) nor a “systematic review of systematic reviews”—a systematic review addresses a clinically-based research question, ideally based on PICO. The cited paper is an overview of systematic reviews.
6. The point about lack of registration in PROSPERO is a valid one, according to Sarkis-Onofre *et al.,* since it complies with the instructions of PROSPERO. However, all studies that have an a priori protocol can register it (and benefit from this) and many different appropriate repositories exist.
7. I’m not sure what is the point about the protocol being mentioned but not given. It doesn’t matter what the authors say: if there is no publicly available registered/time-stamped protocol, there is none.
8. Likewise, I’m not sure what is meant with the 3 ^rd^ paragraph. Information provided through e-mails might not necessarily be regarding the same way as published information, but can still be used in a scientific publication, if deemed appropriate.
9. The paragraph about the Sarkis-Onofre *et al. *authors wrongly judging some AMSTAR 2 fields of your SR seem to be valid, however.
10. Likewise, the comment about potentially missed SRs to include seems to be valid.
11. The last bullet point might not necessarily be correct, since PRISMA is meant for systematic review—it is not a reporting guideline for overviews of review (though some fields of it might be adopted with modifications).

Are arguments sufficiently supported by evidence from the published literature or by new data and results?

No

Is the conclusion balanced and justified on the basis of the presented arguments?

No

Is the rationale for commenting on the previous publication clearly described?

Yes

Are any opinions stated well-argued, clear and cogent?

Partly

Reviewer Expertise:

orthodontics; evidence-based dentisty; systematic reviews

I confirm that I have read this submission and believe that I have an appropriate level of expertise to state that I do not consider it to be of an acceptable scientific standard, for reasons outlined above.

# Response

**Published:** 2021-11-16

**Authors:** Kelvin Afrashtehfar

**Affiliations:** Ajman University, United Arab Emirates

Dr. Papageorgiou is thanked for his contribution after the correspondence author suggested him as a reviewer.

 The correspondence author is glad to answer his comments and clear any confusion Dr. Papageorgiou has referred to.

 The overview authors will be invited to reply to the correspondence author comment upon completion. As the reviewer may know, some dental journals do not accept letters to the Editor. JERD does not accept replica or letters to the Editor, whereas F1000Research provides the opportunity to have this correspondence open to the public free of charge. Thus, the latter is a different option and could be considered, by some researchers, an ideal journal to allocate the correspondence comment.

 Reply to the main specific comments:

 The correspondence author agrees with Dr. Papageorgiou that methodological quality assessment is not the same as the risk of bias. However, the correspondence author must say that an “overview of systematic reviews” is the same as a “systematic review of systematic reviews.” Kindly refer to Pollock M, Fernandes RM, Becker LA, Pieper D, Hartling L. Chapter V: Overviews of Reviews. In: Higgins JPT, Thomas J, Chandler J, Cumpston M, Li T, Page MJ, Welch VA (editors). Cochrane Handbook for Systematic Reviews of Interventions version 6.2 (updated February 2021). Cochrane, 2021. Available from www.training.cochrane.org/handbook.

 The correspondence author does understand that PROSPERO is probably the most popular repository among many other available options. Indeed, the correspondence author did refer to PROSPERO as the cited paper did. In fact, the correspondence author quoted their statement. The correspondence author appreciates that Dr. Papageorgiou found valid the point concerning the lack of PROSPERO registration.

 The point of mentioning PROSPERO is supported by AMSTAR-2, which considers “protocol registered before start of review” as a critical domain. Please refer to Shea BJ, Reeves BC, Wells G, Thuku M, Hamel C, Moran J, Moher D, Tugwell P, Welch V, Kristjansson E, Henry DA. AMSTAR 2: a critical appraisal tool for systematic reviews that include randomised or non-randomised studies of healthcare interventions, or both. BMJ 2017; 358: j4008. Thus, one would expect that an overview of reviews (or systematic review of systematic reviews) should avoid missing such critical domain that they have assessed in their study where they used systematic reviews as the analytic unit.

 The correspondence author thanks Dr. Papageorgiou for all the other comments that he found valid. For instance, the overview is missing to include SRs, review authors wrongly judging some AMSTAR-2 fields of a previous SR by the correspondence author, and lack of repository registry.

 Lastly, the author appreciates Dr. Papageorgiou's comments regarding this correspondence comment. The points that could be perceived as a disagreement have been addressed by the correspondence author here.

# Peer review: Reviewer response for version 1

**DOI:** 10.5256/f1000research.56469.r92392

**Published:** 2021-10-15

**Authors:** Luca Testarelli, Rodolfo Reda

**Affiliations:** 1 Department of Oral and Maxillofacial Sciences, School of Dentistry, Sapienza University of Rome, Rome, Italy

The evaluations reported in this article are of high quality and demonstrate a great knowledge of the literature and of the analysis of articles present in this article thanks to the reporting items and quality assessments.

 The author underlines in this commentary his criticisms of the review under consideration and makes suggestions for improving the methodology and reporting of this kind of research. The author appears to be involved in Sarkis-Onofre's research, and this limits his field of action in contesting the execution of the study, underlining how another research of his has been excluded. However, we believe that the criticisms raised go beyond the conflict of interest and can be considered valid.

 As described by the author, we agree with the need for more transparency on the methodology of these studies.

 Regarding the registration on PROSPERO, we believe that this kind of revision remains valid regardless of this. The contestation of the protocol used by the author, rather than leading to the use of standardized protocols, we believe is necessary that it be well described in the article and that it be available upon request by the authors of the study.

 The criticisms that are posed in the article, we believe are valid and well structured. The bibliography that underlies them is thick and published in high-level journals. We, therefore, consider this article suitable for indexing, sure of the fact that it can be an added value for the quality of this type of research.

Are arguments sufficiently supported by evidence from the published literature or by new data and results?

Yes

Is the conclusion balanced and justified on the basis of the presented arguments?

Yes

Is the rationale for commenting on the previous publication clearly described?

Yes

Are any opinions stated well-argued, clear and cogent?

Yes

Reviewer Expertise:

Oral Surgery, Periodontics, Endodontics, Restorative Dentistry, CBCT, Imaging in Dentistry.

We confirm that we have read this submission and believe that we have an appropriate level of expertise to confirm that it is of an acceptable scientific standard.

# Response

**Published:** 2021-11-16

**Authors:** Kelvin Afrashtehfar

**Affiliations:** Ajman University, United Arab Emirates

The experienced clinician-scientists and reviewers, Prof. **Luca Testarelli** and Prof. **Rodolfo Reda**, are thanked for finding value and expertise in this correspondence comment.

 The reviewers acknowledged that the criticisms found in this work do not represent a conflict of interest; thus, they were considered valid.

 Interestingly, there was also an agreement between the reviewers and the author for improved transparency on the conduction and reporting of overview studies.

 The reviewers found additional value in having overviews pursuing a formal repository registry and presenting the identification number.

 Lastly, the reviewers are acknowledged for comprehensively appraising this work and supporting the publication of this manuscript.

# Peer review: Reviewer response for version 1

**DOI:** 10.5256/f1000research.56469.r86654

**Published:** 2021-07-21

**Authors:** Marta Roqué-Figuls

**Affiliations:** 1 Iberoamerican Cochrane Centre, Biomedical Research Institute Sant Pau (IIB Sant Pau), Barcelona, Spain

This comment from Afrashtehfar refers to an overview by Sarkis-Onofre *et al.*, 2019 ^1^, published in Esthet Restor Dent, which discusses key aspects of systematic reviews in restorative dentistry, focusing on the improvement of the conduct and reporting of these reviews. The author of the comment identifies specific shortcomings of the Sarkis-Onofre *et al.* overview and formulates some suggestions to improve future methodological quality assessment manuscripts.

 As a general thought, this comment would surely be better suited to be published in the original journal, in order to reach the same audience that read the original paper.

 The author of this comment is also an author of a review included in Sarkis-Onofre *et al*. and illustrates his criticisms of their paper with the assessments made to his review. Given this clear conflict of interest, he should be careful in formulating subjective assessments such as “Perhaps Sarkis-Onofre *et al*. should have dedicated more time to conduct an adequate assessment“, or using the terms ‘safe’ or ‘respectful’.

 The author criticises the methods applied by Sarkis-Onofre *et al.*, particularly that a single researcher conducted the study selection and AMSTAR-2 assessment. These criticisms are based on perceived errors in the AMSTAR-2 assessments made to his own review included in Sarkis-Onofre *et al.,* and on the failure to identify and include in the overview another work he authored (Afrashtehfar *et al*., 2016 ^2^). While these are undeniable facts and the methods applied in the overview were subpar, it’s not clear whether the errors identified are the exception or the norm, nor their impact on the overview conclusions.

 He also points out the need for more transparency in the reporting of AMSTAR-2 assessments whenever these challenge personal communications with the original study authors. I fully support the idea to present in an appendix the clarifications about methodology provided in personal communications and the reasons for Sarkis-Onofre *et al.* to accept or disregard them in each case.

 However, there are two instances of unjustified statements made in the comment. First, the author challenges the justification of Sarkis-Onofre *et al.* for not registering their methodological overview in PROSPERO, arguing that as an overview it could have been registered. However, this fails to acknowledge that methodological overviews that provide no data on patient-relevant outcomes don’t verify PROSPERO registration criteria for methodological reviews.

 Second, the author assumes that the overview, which updates a previously published overview from different authors, should follow the original overview protocol. But, while any review must have a pre-specified protocol, it's not necessary for an update to follow the protocol of the original review, as that protocol may be inexistent or outdated. What is important is that Sarkis-Onofre *et al.* do have a protocol available at request. 

 The author finishes his comment by proposing a list of suggestions for improving future methodological quality assessment manuscripts, mostly already known and common sense. However, the recommendation that AMSTAR-2 be applied by experts in the field rather than research students lacks evidence and is quite debatable.

Are arguments sufficiently supported by evidence from the published literature or by new data and results?

Partly

Is the conclusion balanced and justified on the basis of the presented arguments?

Partly

Is the rationale for commenting on the previous publication clearly described?

Yes

Are any opinions stated well-argued, clear and cogent?

Partly

Reviewer Expertise:

I am a statistician, author of several systematic reviews, and an editor in two Cochrane Review Groups, conducting peer reviews frequently.

I confirm that I have read this submission and believe that I have an appropriate level of expertise to confirm that it is of an acceptable scientific standard, however I have significant reservations, as outlined above.

## References

[1] : Systematic reviews in restorative dentistry: discussing relevant aspects. *J Esthet Restor Dent* .31(3) : 10.1111/jerd.12463 222-232 10.1111/jerd.12463 30821908

[2] : Failure of single-unit restorations on root filled posterior teeth: a systematic review. *Int Endod J* .2017;50(10) : 10.1111/iej.12723 951-966 10.1111/iej.12723 27870102

# Response

**Published:** 2021-11-16

**Authors:** Kelvin Afrashtehfar

**Affiliations:** Ajman University, United Arab Emirates

I thank Prof. Roqué-Figuls for supporting the idea of presenting an appendix with methodological clarifications provided in the e-mail exchange between the overview authors (Sarkis-Onofre R, Pereira-Cenci T, Tricco AC, Demarco FF, **Moher D**, Cenci MS. Systematic reviews in restorative dentistry: discussing relevant aspects. J Esthet Restor Dent. 2019 May;31(3):222-232) and the comment author.

 Regarding the overview protocol registration, AMSTAR-2 considers “protocol registered before start of review” as a critical domain. Please refer to Shea BJ, Reeves BC, Wells G, Thuku M, Hamel C, Moran J, **Moher D**, Tugwell P, Welch V, Kristjansson E, Henry DA. AMSTAR 2: a critical appraisal tool for systematic reviews that include randomised or non-randomised studies of healthcare interventions, or both. BMJ 2017; 358: j4008. Thus, one would expect that an overview of reviews (or systematic review of systematic reviews) should avoid missing such critical domain that they have assessed in their study where they used systematic reviews as the analytic unit.

 Indeed, the author does defend the statement that experienced researchers ideally should apply AMSTAR2 instead of inexperienced research students.

 Prof. Roqué-Figuls is thanked for expressing her opinions. However, the comment author did not realize of the reviewer's previous participation in the following publications:

- Sáenz Calvo A, Fernández Esteban I, Mataix Sanjuán A, Ausejo Segura M, Roqué M, **Moher D**. [Metformin for type-2 diabetes mellitus. Systematic review and meta-analysis]. Aten Primaria. 2005 Sep 15;36(4):183-91. 
- Saenz A, Fernandez-Esteban I, Mataix A, Ausejo M, Roque M, **Moher D**. Metformin monotherapy for type 2 diabetes mellitus. Cochrane Database Syst Rev. 2005 Jul 20;(3):CD002966. 

The author accepts being concerned and kindly requests the reviewer to reconsider her decision about this comment. 

 The comment author has complied with answering the initial reviewer's comments. Lastly, after this participation, the author has no more comments or replies.
